# Vertebral Osteomyelitis, Discitis, and Epidural Abscess: A Rare Complication of *Cardiobacterium* Endocarditis

**DOI:** 10.1177/2324709618807504

**Published:** 2018-10-28

**Authors:** Sanjay K. Yadava, Ambika Eranki

**Affiliations:** 1 State University of New York Upstate Medical University, Syracuse, NY, USA

**Keywords:** *Cardiobacterium*, endocarditis, osteomyelitis

## Abstract

In this article, we report the case of a 75-year-old man who was presented with new low back pain for 2 weeks. His past history was significant for severe aortic stenosis necessitating bioprosthetic aortic valve placement 4 years ago, hypertension, and coronary artery disease. His physical examination was positive for point tenderness over the lower lumbar spine. He was found to be bacteremic with *Cardiobacterium hominis*. Magnetic resonance imaging of the spine showed lumbar (L4-L5) epidural abscess and vertebral osteomyelitis, discitis. He underwent a computed tomography–guided needle biopsy of L4-L5. The biopsy culture was also positive for *Cardiobacterium hominis*. A transesophageal echocardiogram showed small vegetation on the mitral valve with mild regurgitation. He was started on intravenous ceftriaxone 2 g once daily for a planned duration of 6 weeks and was discharged. However, he, unfortunately, expired at an outside facility secondary to an unknown illness 4 weeks into the treatment course.

## Introduction

*Cardiobacterium* is a member of HACEK group (*Haemophilus paraphrophilus, Haemophilus parainfluenzae, Aggregatibacter actinomycetemcomitans, Aggregatibacter aphrophilus, Cardiobacterium hominis, Eikenella corrodens*, and *Kingella kingae*), which is known to be the cause of endocarditis but rarely associated with other infections. Only 2 cases of discitis caused by the organism have been reported in the English literature.

## Case Presentation

A 75-year-old man presented with dull aching new-onset low back pain for 2 weeks. His past history was significant for severe aortic stenosis necessitating bioprosthetic aortic valve placement 4 years ago, hypertension, and coronary artery disease. His physical examination was positive for point tenderness over the lower lumbar spine. At presentation, he had a fever of 38.7°C, heart rate of 96/min, blood pressure of 130/90 mm Hg, and oxygen saturation of 96% on room air. On physical examination, tenderness over lower lumber vertebra noted without deformity, skin lesion, or focal neurological deficit. A new holosystolic murmur was also noted at the mitral area.

**Figure 1.**
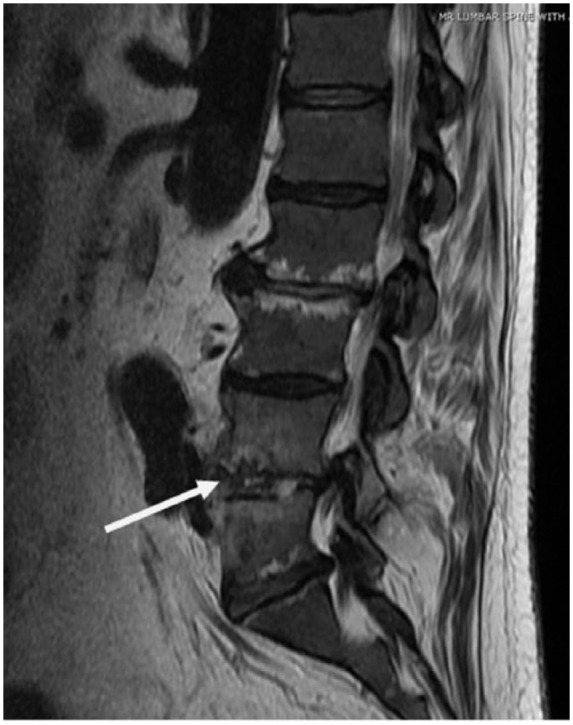
Magnetic resonance imaging: Discitis/osteomyelitis at L4-L5 with preservation of vertebral body height but an extension of infection into the epidural space, as well as anteriorly and into the left posterior paraspinal soft tissues.

**Figure 2.**
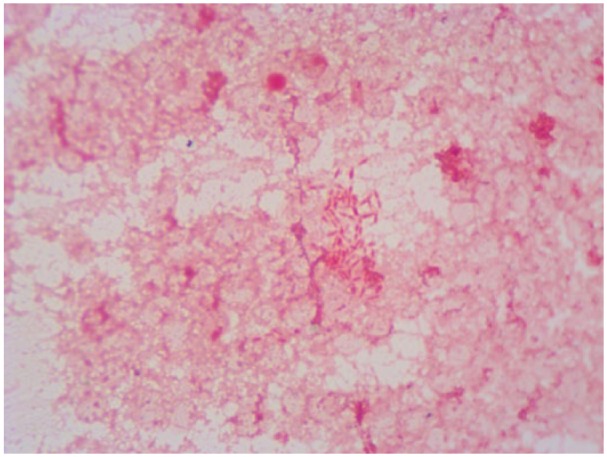
Gram staining of vertebral biopsy, gram-negative rods.

His white blood cell count was 4.33 × 10^3^/µL (normal = 4-10 × 10^3^/µL), hemoglobin/hematocrit of 6.8 g/dL/20.6%, and thrombocytopenic to 100 × 10^3^/µL (normal = 150-400 × 10^3^/µL) with normal renal and liver function tests. His erythrocyte sedimentation rate and C-reactive protein were elevated to 107 mm/h and 205 mg/L, respectively. Magnetic resonance imaging of the spine revealed lumbar (L4-L5) epidural abscess and vertebral osteomyelitis, discitis (Figure 1). He was found to be bacteremic with *C hominis*. He underwent a computed tomography–guided needle biopsy of L4-L5. The biopsy culture was also positive for *C hominis* (Figure 2). A transesophageal echocardiogram showed small vegetation on the mitral valve with mild regurgitation. He was started on intravenous ceftriaxone 2 g once daily for a planned duration of 6 weeks and was discharged. However, he, unfortunately, expired at an outside facility secondary to an unknown illness 4 weeks into the treatment course.

## Discussion

*Cardiobacterium hominis* is a slow-growing, fastidious, capnophilic, gram-negative bacillus that is a member of the HACEK group.^1,2^ It commonly colonizes the human oropharynx as normal indigenous flora and may be rarely found on another mucosal surface. There are only 2 species in the genus *Cardiobacterium: C hominis and C valvarium*. They are frequently bulbous at one or both ends and arranged in short chains or rosettes. These organisms rarely cause disease other than endovascular infection. Human infections are endogenously acquired. Risk factors for *Cardiobacterium* infection include the following: poor dentition, recent dental procedure, prior endocarditis, cardiac anatomical abnormalities (rheumatic heart disease, ventricular septal defect, and congenital bicuspid valve), recent endoscopy, cardiac implantable electronic device, and prosthetic heart valve.^3^ Our patient had no any risk factor other than the prosthetic valve.

*Cardiobacterium* is a known but rare cause of subacute native, prosthetic valve and cardiac implantable electronic device endocarditis.^3,4^ Moreover, septic arthritis, pericarditis, bacterial meningitis, osteomyelitis, neonatal sepsis, peritonitis, and lacrimal gland infection have also been reported in addition to endovascular infections.^5-11^ *Cardiobacterium* species were shown to be part of the plaques in smokers in a study investigating marginal and subgingival plaque formation.^12^ To the best of our knowledge, only 2 cases of discitis caused by this organism have been reported in English literature.^10,13^

Infection due to *Cardiobacterium* usually has a subacute presentation with insidious onset leading to delay in diagnosis. Some patients may have anemia, splenomegaly, and immune-mediated glomerulonephritis on presentation.^3^ Our patient had chronic anemia, thrombocytopenia of undetermined etiology, which worsened with current infection requiring blood transfusion. Given the chronicity of anemia, thrombocytopenia, and poor response to infection, there was a strong suspicion of myelodysplastic syndrome; unfortunately, the patient passed away without a definitive diagnosis. The anemia and thrombocytopenia of our patient slightly improved with treatment; however, it never normalized and there was no clinical or laboratory evidence of splenomegaly or glomerulonephritis. Due to delay in presentation, large vegetation and large vessel emboli are characteristics of endocarditis caused by this organism.

These organisms grow slowly in standard blood culture media, and recovery may need prolonged incubation, 2 to 3 weeks. Traditionally, the microbiology laboratory needs to be notified to retain blood culture for 2 weeks or longer in patients who had had a suspicion of endocarditis by these organisms. Now, with modern automated blood culture detection system and media, these organisms can be detected within 3 to 5 days of incubation.^14^

Sometimes *Cardiobacterium* is misidentified as *Pasteurella multocida*, as both are catalase-negative and oxidase-positive gram-negative rods. However, thanks to a recent development in diagnostic microbiology with the use of MALDI-TOF (matrix-assisted laser desorption ionization time-of-flight mass spectrometry), diagnosis is not only faster and more reliable, but it is also more accurate. Nucleic acid amplification and using 16S ribosomal RNA gene sequence analysis from tissue (heart valve) is useful in the identification of culture-negative infection.

Susceptibility testing is usually not performed because of the slow growth of the organism and unusual nutritional requirements. Cardiobacterium strains are almost always sensitive to penicillins and other β-lactams, as well as aminoglycosides and quinolones when tested. Ceftriaxone for 4 weeks in native valve endocarditis and 6 weeks in prosthetic valve endocarditis is recommended by the Infectious Diseases Society of America guideline as a preferred therapy.^15^ In our patient, even though there was no visible vegetation on the prosthetic valve by transesophageal echocardiogram, given the bacteremic status of the patient in the presence of a prosthetic aortic valve, the likelihood of infection of the prosthetic valve was high. Considering bone infection and possible prosthetic valve infective endocarditis, we decided to treat with 6 weeks of antibiotic to our patient. Ampicillin may be an option if there is in vitro susceptibility. In patients who cannot tolerate ampicillin or cephalosporins, quinolones can be used.

In conclusion, infectious disease physicians must have a high index of suspicion for metastatic foci of infection in patients with HACEK endocarditis, as this leads to earlier diagnosis and appropriate therapy.
